# LncRNA NEF inhibits migration and invasion of HPV-negative cervical squamous cell carcinoma by inhibiting TGF-β pathway

**DOI:** 10.1042/BSR20180878

**Published:** 2019-04-26

**Authors:** Wencui Ju, Xiaoyong Luo, Nan Zhang

**Affiliations:** Department of Oncology, Luoyang Center Hospital Affiliated Zhengzhou University, Louyang City, Henan Province, 471000, PR China

**Keywords:** cervical squamous cell carcinoma, lncRNA NEF, HPV, TGF-β1

## Abstract

LncRNA NEF was a recently identified tumor suppressor lncRNA in hepatocellular carcinoma. Our study aimed to explore the role of NEF in cervical squamous cell carcinoma (CSCC) patients. In the present study, expression of NEF in tumor tissue (cervical biopsies for healthy control) and serum of human papillomaviruses (HPV)-negative and HPV-positive CSCC patients as well as healthy controls was detected by qRT-PCR. Diagnostic and prognostic values of NEF for CSCC were evaluated by ROC curve and survival curve analysis, respectively. NEF expression vector was transfected into CSCC cells and the effects on cell migration and invasion as well as TGF-β1 expression were investigated by Transwell migration assay, Transwell invasion assay, and Western blot, respectively. We found that expression of NEF in cervical tissues (tumor tissues for CSCC patients) and serum was significantly down-regulated in HPV-negative CSCC patients than in healthy controls and HPV positive patients, but no significant differences were found between healthy controls and HPV positive patients. Low serum levels of NEF distinguished HPV-negative CSCC patients from healthy controls and indicated poor survival. NEF overexpression inhibited the migration and invasion of HPV-negative but not HPV-positive CSCC cells. NEF overexpression down-regulated TGF-β1 in HPV-negative CSCC cells but not in HPV-positive CSCC cells. TGF-β1 treatment reduced the effects of NEF overexpression on cell migration and invasion. Therefore, we conclude that lncRNA NEF may inhibit the migration and invasion of HPV-negative cervical squamous cell carcinoma by inhibiting TGF-β pathway.

## Introduction

Cervical cancer is a major burden of cancer worldwide [1]. Approximately 90% of cervical cancers are caused by human papillomaviruses (HPV) infection [2,3]. With advances in understanding the pathogenesis of cervical cancer, correlations with HPV genotypes and the occurrence of cervical cancer have been elucidated in details [4]. Popularization of HPV infection screening and increasing HPV vaccination rate has significantly reduced the incidence of cervical cancer during 20th century [2–4]. However, no significant reduction in incidence of cervical cancer is been observed during last decade [5,6]. Besides HPV infection, cervical cancer can also be caused by other factors. Poor prognosis is usually observed in patients with HPV-negative cervical cancer and incidence of HPV-negative cervical cancer also shows an increasing trend in recent years [6].

Cervical cancer is divided into cervical squamous cell carcinoma (CSCC) and cervical adenocarcinoma, two subtypes [7]. TGF-β signaling pathway plays pivotal roles in the development and progression of various types of human malignancies [8]. As a double-edge sword in cancer biology, TGF-β signaling shows both inhibitor effects on tumor growth and enhancing effects on tumor metastasis [9]. LncRNAs are a subgroup of non-coding RNAs that composed of more than 200 nt participates in nearly all critical aspects of the onset, development and progression of different types of tumors [10]. The cross-talk between TGF-β signaling and lncRNAs has been widely investigated in different disease models [11]. NEF is a novel lncRNA with characterized functionality only in hepatocellular carcinoma [12]. In our study, we observed that NEF may inhibit HPV-negative CSCC metastasis by down-regulating TGF-β1 expression.

## Materials and methods

### Specimen collection

Tumor tissues and serum samples of 48 HPV-negative CSCC patients and 52 HPV-positive patients (19 HPV: 11 cases, 21 HPV: 16 cases and 12 HPV: 18 cases) were obtained from specimen library of Luoyang Center Hospital affiliated Zhengzhou University. Those patients were diagnosed treated in Luoyang center hospital affiliated Zhengzhou University from July 2010 to July 2012. All patients were diagnosed and treated for the first time. All those patients completed treatment and follow-up. Patients infected with other viruses or with other type of malignancies or other severe diseases, or patients died of other reasons during follow-up were excluded from the present study. The 48 HPV-negative CSCC patients had a mean age of 48.4 ± 5.6 year (ranged from 33 to 69 years), while the mean age of HPV-negative CSCC patients was 47.9 ± 5.3 year (ranged from 30 to 68 years). Cervical tissues and serum samples of 38 healthy females who received routine physiological examinations during the same time period were also obtained from specimen library of Luoyang Center Hospital affiliated Zhengzhou University to serve as control group. Those healthy females received cervical biopsies for the detection of potential cervical lesions, but cervical diseases were finally excluded. Control group had a mean age of 47.8 ± 4.9 year (ranged from 34 to 65 years). No significant differences in age, BMI and other basic clinical data were found between HPV-negative CSCC patients, HPV-positive CSCC patients and control group. This research has been carried out in accordance with the World Medical Association Declaration of Helsinki, and that all subjects provided written informed consent.

### Real-time quantitative reverse transcription PCR

All total RNA extractions were performed using Trizol reagent (Invitrogen, U.S.A.). Tissues were ground in liquid nitrogen before adding Trizol reagent. cDNA was synthesized through reverse transcription and 20 μl PCR reaction systems were prepared using SYBR® Green Real-Time PCR Master Mixes (Thermo Fisher Scientific, U.S.A.). Primers used in PCR were: 5′-CTGCCGTCTTAAACCAACCC-3′ (upstream) and 5′-GCCCAAACAGCTCCTCAATT-3′(downstream) for human lncRNA-NEF; 5′-GACCTCTATGCCAACACAGT-3′ (upstream) and 5′-AGTACTTGCGCTCAGGAGGA-3′ (downstream) for β-actin. PCR reaction conditions were: 95°C for 1 min 20 s, followed by 40 cycles of 10 s at 95°C and 30 s at 57°C. Expression of NEF was normalized to β-actin according to 2^−ΔΔ*C*^_T_ method.

### Cell lines, cell culture and transfection

HPV-negative human cervical squamous cell carcinoma cell line, C33A, and HPV-positive human cervical squamous cell carcinoma cell line, SiHa, were purchased from ATCC. Cells of those two cell lines were cultured according to the instructions provided by ATCC in an incubator (37°C, 5% CO_2_). Full-length NEF cDNA surrounded by EcoRI-EcoRI cutting sites were amplified by PCR. This fragment was linked to linearized pIRSE2-EGFP vector (Clontech, Palo Alto, CA, U.S.A.) to make NEF expression vector. NEF siRNA and negative control siRNA were synthesized by Sangon (Shanghai, China). NEF expression vectors or empty pIRSE2-EGFP vectors (negative control) were transfected into 5 × 10^5^ cells at a concentration of 15 nM using Lipofectamine 2000 reagent (11668-019, Invitrogen, Carlsbad, U.S.A.) according to the instructions of the kit. For NEF siRNA and negative control siRNA transfection, the dose was 40 nM. Expression of NEF was checked by qRT-PCR just before subsequent experiments to make sure an overexpression rate no <150% was reached.

### *In vitro* cell migration and invasion assay

After transfection and confirmation of overexpression, cells were collected and cell suspension with as cell density of 5 × 10^4^ cells per ml was prepared using Dulbecco’s modified Eagle’s Medium containing 1% fetal calf serum (FCS; Gibco; Thermo Fisher Scientific, Inc.). Then, 0.1 ml of cell suspension containing 5 × 10^3^ cells was added into the upper chamber, while the lower chamber was filled with Dulbecco’s modified Eagle’s Medium containing 10% FCS. After incubation for 24 h, invading cells on membranes were stained with 1% Crystal Violet (Sigma-Aldrich; Merck KGaA) at 25˚C for 30 min. Stained membranes were observed under a light microscope (magnification, ×100) and cells were counted. Transwell invasion chambers pre-coated with Matrigel (50 μl/filter; BD Biosciences, Franklin Lakes, NJ, U.S.A.) was used in invasion assay according to the manufacturer’s instructions, and all other steps are essentially the same of migration assay.

### Western blot

All total protein extractions were performed using RIPA solution (Thermo Fisher Scientific, U.S.A.), and protein concentrations were measured using BSA method. After that SDS-PAGE (12%) gel electrophoresis was performed with 35 µg protein per lane. Following gel transfer, PVDF membranes were incubated with 5% skimmed milk at room temperature for 2.5 h. Membranes were then incubated with primary antibodies of TGF-β1 (rabbit anti human, 1:1400, ab50716, Abcam) and GAPDH (rabbit anti human, 1:1200, ab37168, Abcam) overnight at 4°C. After complete washing with TBST (0.3% Tween 20), membranes were further incubated with goat anti-rabbit IgG-HRP secondary antibody (1:1000, MBS435036, MyBioSource) at room temperature for 4 h. Pierce ECL Western Blotting Substrate (Thermo Scientific) was dropped onto membranes to develop signals, and ImageJ software was used to normalize expression of TGF-β1 to endogenous control GAPDH.

### Data analysis

SPSS19.0 (SPSS Inc., U.S.A.) statistical software was used for data analysis. Correlations between NEF expression and patients’ clinicopathological data were analyzed by Chi square test. Protein and mRNA expression, as well as cell migration and invasion data were compared by one-way analysis of variance followed by LSD test. ROC curve analysis was performed to evaluate the diagnostic value of NEF expression for HPV-negative CSCC with HPV-negative CSCC patients as true cases and healthy controls as true negative cases. Thirty HPV-negative CSCC patient were divided into high and low expression group, fifteen in each group. Survival curves were plotted and compared by K-M method and log rank *t-*test, respectively. *P*<0.05 indicated a difference with statistical significance.

## Results

### Expression of NEF in cervical tissues of CSCC patients and healthy controls

**Figure 1:**
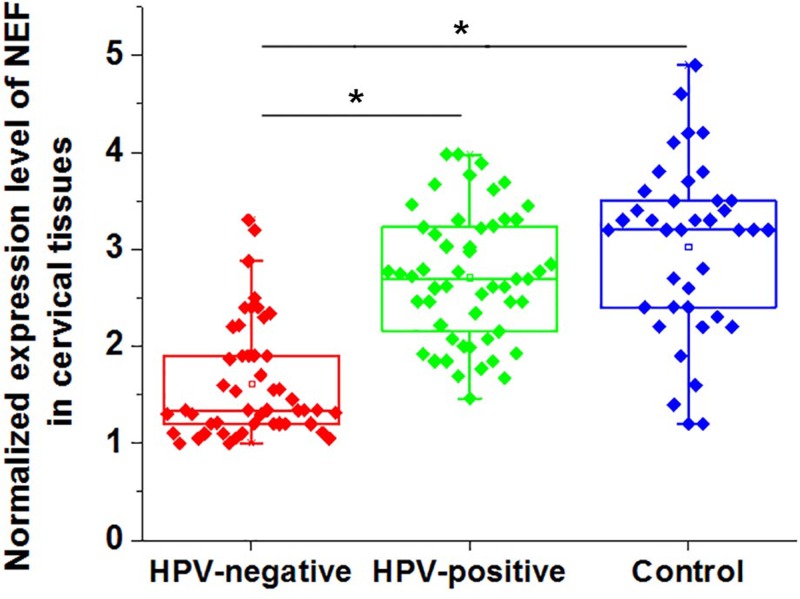
Expression of NEF in cervical tissues of CSCC patients and healthy controls Notes: *P<0.05.

Expression of NEF in tumor tissues of 48 HPV-negative CSCC patients and 52 HPV-positive patients and in cervical biopsies of 38 healthy females was detected by qRT-PCR. As showed in Figure 1, expression of NEF in cervical tissues was significantly down-regulated in HPV-negative patients than in HPV-positive patients and healthy controls (*P*<0.05). In addition, no significant differences in NEF expression was found between HPV-positive patients and healthy controls (*P*>0.05).

### Comparison of serum levels of NEF in between CSCC patients and healthy controls

**Figure 2:**
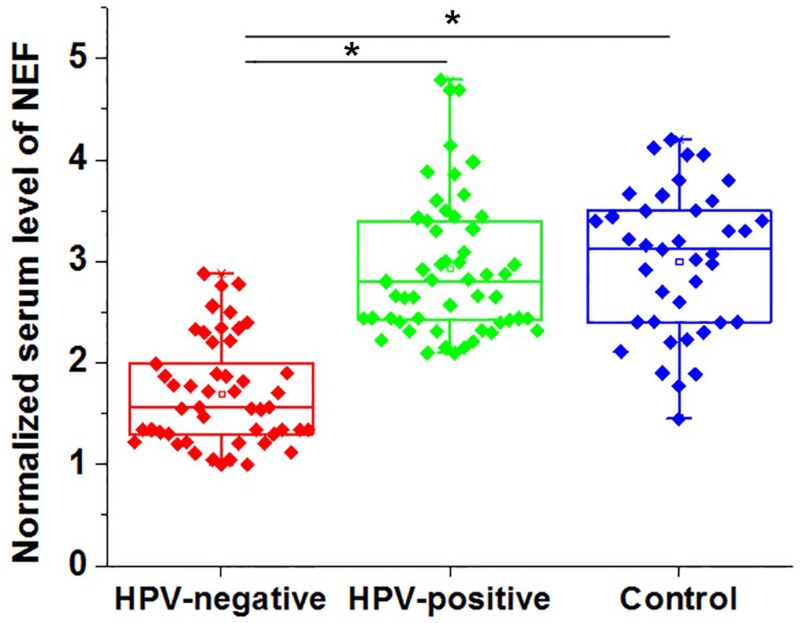
Comparison of serum levels of NEF in between CSCC patients and healthy controls *, *P*<0.05.

Serum levels of NEF in 48 HPV-negative CSCC patients, 52 HPV-positive patients and 38 healthy females were also measured by qRT-PCR. As showed in Figure 2, serum levels of NEF were significantly lower in HPV-negative patients than in HPV-positive patients and healthy controls (*P*<0.05). In addition, no significant differences in serum levels of NEF were found between HPV-positive patients and healthy controls (*P*>0.05).

### Diagnostic values of NEF expression for HPV-negative CSCC

**Figure 3:**
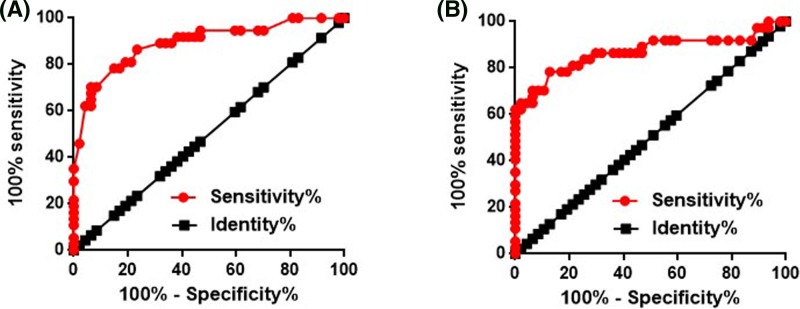
Diagnostic values of NEF expression for HPV-negative CSCC The diagnostic values of NEF expression in cervical tissues (**A**) and serum (**B**) for HPV-negative CSCC.

In view of the differential expression of NEF in HPV-negative CSCC patients and healthy control, ROC curve analysis was performed to evaluate the diagnostic value of NEF expression for HPV-negative CSCC. For NEF expression in cervical tissues, the area under the curve (AUC) was 0.8910 with standard error of 0.03656 and 95% confidence interval of 0.8193 to 0.9627 (Figure 3A). For serum NEF, AUC was 0.8677 with standard error of 0.04484 and 95% confidence interval of 0.7798 to 0.9556 (Figure 3B).

### Correlation between NEF expression and clinicopathological data of patients with HPV-negative CSCC

Chi square analysis was performed to analyze the correlations between NEF expression levels and clinicopathological data of patients with HPV-negative CSCC. As shown in Tables 1 and 2, expression levels of NEF in cervical tissues and serum showed no significant correlations with patients’ age and tumor size (*P*>0.05), but were significantly correlated with distant tumor metastasis (*P*<0.05).

**Table 1:** Correlation between NEF expression levels in cervical tissues and clinicopathological data of patients with HPV-negative CSCC

Items	Groups	Cases	High-expression	Low-expression	χ²	p value
Age	>50(years)	22	9	13	1.34	0.25
	<50 (years)	26	15	11		
Primary tumor diameter	>5 cm	14	8	6	1.42	0.49
	3-5 cm	16	9	7		
	1-3 cm	18	7	11		
Tumor distant metastasis	Yes	30	9	21	12.80	p<0.01
	No	18	15	3		

**Table 2:** Correlation between serum levels of NEF and clinicopathological data of patients with HPV-negative CSCC

Items	Groups	Cases	High-expression	Low-expression	χ²	p value
Age	>50(years)	22	10	12	0.67	0.41
	<50 (years)	26	16	12		
Primary tumor diameter	>5 cm	14	7	7	0.47	0.49
	3-5 cm	16	9	7		
	1-3 cm	18	8	10		
Tumor distant metastasis	Yes	30	9	21	12.80	p<0.01
	No	18	15	3		

### Prognostic values of NEF expression for HPV-negative CSCC with distant tumor metastasis

**Figure 4:**
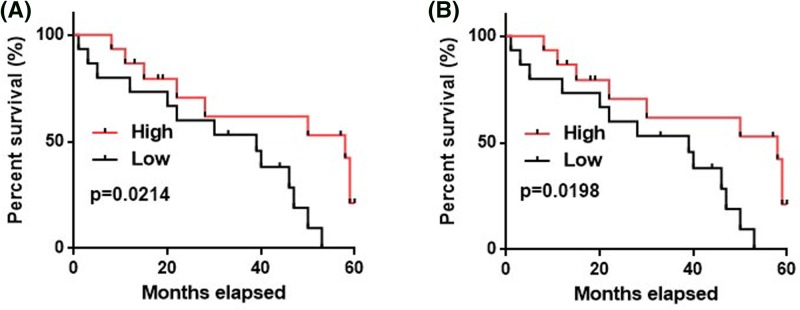
Prognostic values of NEF expression for HPV-negative CSCC with distant tumor metastasis A comparison of survival curves of HPV-negative CSCC patients with high- and low-expression level of NEF in cervical tissues (**A**) and serum (**B**).

The above-mentioned data showed that NEF expression is affected by distant tumor metastasis, which is a major cause of poor prognosis of CSCC patients. Our study included 30 HPV-negative CSCC patients with distant tumor metastasis. According to the medium expression level of NEF in tumor tissues or serum, those patients were divided into high- and low-expression group, 15 in each group. Those patients were followed-up for 5 years and their overall survival conditions were recorded. Kaplan–Meier method was used to plot survival curves for both groups, followed by comparison of survival curves using log rank *t* test. As shown in Figure 4, overall survival of patient with low-expression level of NEF in cervical tissues (Figure 4A,B) was significantly worse than those with high-expression level of NEF.

### Effects of NEF overexpression and siRNA silencing on TGF-β1 expression in HPV-negative and HPV-positive CSCC cells

**Figure 5:**
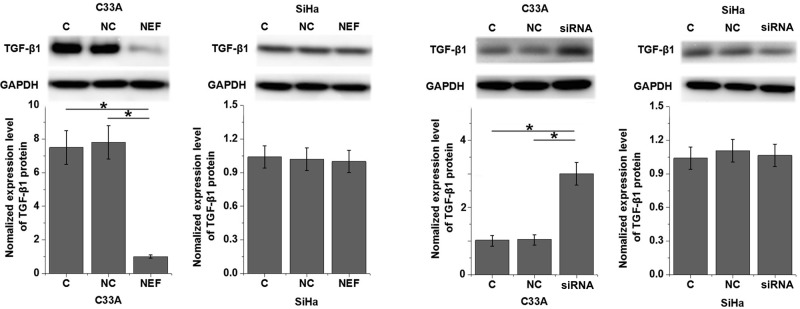
Effects of NEF overexpression and siRNA silencing on TGF-β1 expression in HPV-negative and HPV-positive CSCC cells *P < 0.05.

TGF-β1 plays pivotal roles in the metastasis of various types of malignancies, and inhibition of TGF-β1 is considered to be a promising target for the treatment of cervical cancer [13]. In the present study, expression of TGF-β1 in cells of HPV-negative human cervical squamous cell carcinoma cell line, C33A, and HPV-positive human cervical squamous cell carcinoma cell line, SiHa, was detected by Western blot after transfection of NEF expression vector. As shown in Figure 5, NEF overexpression significantly down-regulated, while NEF siRNA silencing significantly up-regulated the expression of TGF-β1 in cells of human cervical squamous cell carcinoma cell line, C33A (*P*<0.05), but not in HPV-positive human cervical squamous cell carcinoma cell line, SiHa. In addition, TGF-β1 (10 ng/ml, Sigma-Aldrich) treatment showed no significant effects on NEF expression (data not shown).

### Effects of NEF overexpression and TGF-β1 treatment on migration and invasion of HPV-negative and HPV-positive CSCC cells

**Figure 6:**
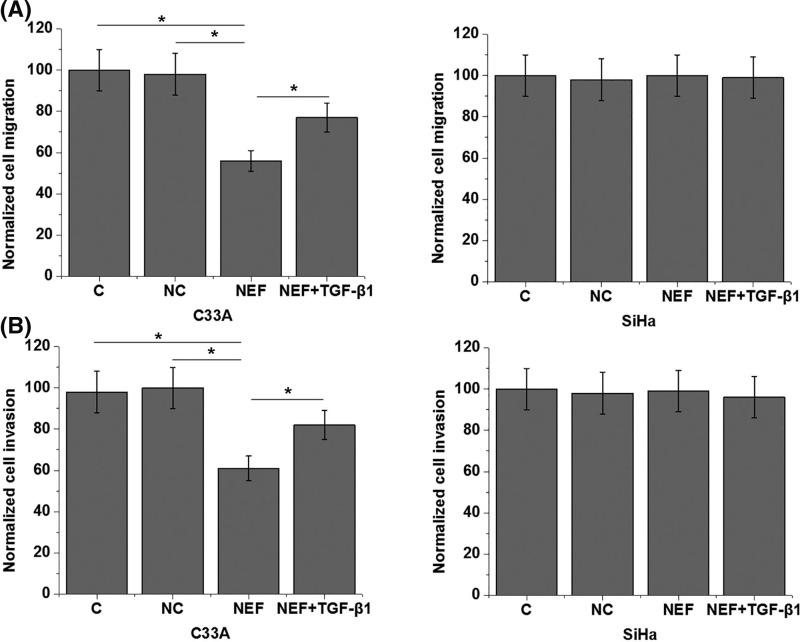
Effects of NEF overexpression and TGF-β1 treatment on migration and invasion of HPV-negative and HPV-positive CSCC cells The effects of NEF overexpression and TGF-β1 treatment on migration (**A**) and invasion (**B**) of HPV-negative and HPV-positive CSCC cells; *P<0.05.

As shown in Figure 6A, NEF overexpression significantly promoted the migration of cells of HPV-negative C33A cell line (*P*<0.05), but not cells of HPV-positive SiHa cells (*P*>0.05). Similarly, NEF overexpression also significantly promoted the invasion (Figure 6B) of cells of HPV-negative C33A cell line (*P*<0.05), but not cells of HPV-positive SiHa cells (*P*>0.05). In addition, TGF-β1 (10 ng/ml, Sigma-Aldrich) treatment significantly reduced the effects of NEF overexpression on migration and invasion of cells of HPV-negative cell line, C33A (*P*<0.05).

## Discussion

The key finding of our study is that NEF as a newly discovered tumor suppressor lncRNA in hepatocellular carcinoma [12] may also inhibit the metastasis of HPV-negative CSCC but not HPV-positive CSCC. The action of NEF in HPV-negative CSCC is likely achieved through the down-regulation of TGF-β1.

HPV-negative and -positive CSCC have different pathogenesis, and they should be treated differently [14]. It has been reported that Oct4 as a transcription factor is specifically up-regulated in HPV-positive but not HPV-negative CSCC cell lines [15], indicating that Oct4 may participate in the pathogenesis of CSCC through a HPV-dependent pathway. HPV-negative CSCC is an extremely aggressive malignancy and the prognosis is generally poor [16]. Therefore, specific treatment targets are always needed to improve treatment outcomes of this disease. NEF as a newly discovered lncRNA is down-regulated in hepatocellular carcinoma [12]. Based on our knowledge, expression pattern of this lncRNA in other diseases is still unknown. Results of our study showed that, compared with healthy controls, expression levels of lncRNA NEF were specifically reduced in HPV-negative CSCC but not in HPV-positive CSCC. Therefore, down-regulation of lncRNA NEF is specifically involved in the pathogenesis of HPV-negative CSCC, and NEF may serve as a potential therapeutic target for the treatment of this disease.

Tumor metastasis is still a main challenge for the treatment of CSCC, and early and accurate diagnosis is still the key for patients’ survival [17]. With the advantages of less invasive operation, detection of circulating biomarkers has been widely used in the diagnosis of human diseases [18]. Our study showed that NEF is differentially expressed in HPV-negative CSCC patients and healthy females in both cervical tissues and serum. Therefore, ROC curve analysis was performed to evaluate the diagnostic value of NEF expression for HPV-negative CSCC, and results showed that reduced expression levels of NEF in cervical tissues and serum can be used to effectively distinguish HPV-negative CSCC patients from healthy people. Therefore, NEF may serve as a potential diagnostic marker for HPV-negative CSCC. In addition, detection of serum NEF may be used if biopsy is not applicable.

Correlation analysis showed that NEF expression was significantly correlated with distant tumor metastasis but not tumor size, which is consistent with the role of NEF in hepatocellular carcinoma. Tumor metastasis is a major cause of death in CSCC patients and the survival of CSCC patients at early stages is generally satisfactory [17]. Therefore, in order to exclude the effects of distant tumor metastasis, we only analyzed the follow-up data of 30 HPV-negative CSCC patients with distant tumor metastasis. Results showed that low NEF expression levels in cervical tissues and serum are closely correlated with poor post-operative survival, indicating that NEF may serve as a potential prognostic marker for HPV-negative CSCC.

Our data also showed that NEF is likely an inhibitor of the migration and invasion of HPV-negative CSCC cells but not HPV-positive CSCC cells. Although the role of TGF-β in regulating cancer cell proliferation is controversial, it is generally believed that TGF-β signaling promotes tumor metastasis [19]. In our study, NEF inhibited the expression of TGF-β1 in HPV-negative CSCC cells but not in HPV-positive CSCC cells. In addition, TGF-β1 treatment showed no significant effects on NEF expression but significantly reduced the effects of NEF overexpression on cell migration and invasion. Therefore, NEF may inhibit the migration and invasion of HPV-negative CSCC cells by serving as an upstream inhibitor of TGF-β signaling.

However, molecular mechanism of the regulation of TGF-β signaling by NEF is still unknown. It has been reported that NEF can cis-regulate FOXA2 to antagonize epithelial-to-mesenchymal transition (EMT) [12], and FOXA2 is likely involved in TGF-β1-mediated EMT [20]. Therefore, FOXA2 may be a mediator between TGF-β signaling and NEF.

In conclusion, expression of NEF was specifically down-regulated in HPV-negative CSCC patients but not in HPV positive patients. Low serum levels of NEF may serve a potential diagnostic and prognostic biomarker for HPV-negative CSCC. NEF may inhibit the migration and invasion of HPV-negative but not HPV-positive CSCC cells by down-regulating TGF-β1 expression.

## Availability of data and materials

All data generated or analyzed during this study are included in this published article.

## Abbreviations

CSCC: cervical squamous cell carcinoma
EMT: epithelial-to-mesenchymal transition
GAPDH: glyceraldehyde-3-phosphate dehydrogenase
HPV: human papillomaviruses
PVDF: polyvinylidene fluoride
qRT-PCR: quantitative real time polymerase chain reaction
ROC: receiver operating characteristic curve
TGF-B: transforming growth factor-β
